# Isolated Multifocal Superior Mesenteric Artery Vasculitis With Coexisting Superior Mesenteric Vein Thrombosis: A Unique Coincidence

**DOI:** 10.7759/cureus.18706

**Published:** 2021-10-12

**Authors:** Sai S Allu, Kiranmaye Tiriveedhi

**Affiliations:** 1 Medicine, Warren Alpert Medical School, Providence, USA; 2 Medicine, Mercy Hospital St. Louis, St. Louis, USA; 3 Gastroenterology, Mercy Hospital St. Louis, St. Louis, USA

**Keywords:** superior mesenteric vein, intestinal ischemia, superior mesenteric artery, thrombosis, vasculitis

## Abstract

Vasculitis involving the gastrointestinal (GI) tract typically occurs in association with multisystem disease. Isolated superior mesenteric artery (SMA) vasculitis is a rare disorder that has a high degree of morbidity and mortality. Patients often present with nonspecific symptoms, and this condition can lead to varying degrees of intestinal ischemia, significant gastrointestinal bleeding, and bowel infarction, leading to perforation, peritonitis, and sepsis from bacterial translocation. Diagnosing this condition can be very challenging. High clinical suspicion and early diagnosis using both laboratory workup and appropriate vascular imaging are pivotal in improving outcomes in such patients. Herein, we describe the case of isolated yet multifocal SMA vasculitis with coexisting superior mesenteric vein (SMV) thrombosis. Medical therapy alone resulted in clinical and radiographic improvements. To our knowledge, there have not been any previous reports of this unique coexistence.

## Introduction

Vasculitis involving the gastrointestinal (GI) tract typically occurs in association with multisystem disease. Isolated superior mesenteric artery (SMA) vasculitis is a rare disorder [1]. Although uncommon, this disease has high morbidity and mortality. Patients often present with intestinal ischemia, GI bleeding, and bowel infarction, leading to perforation, peritonitis, and sepsis from bacterial translocation. Timely diagnosis and early management can be very challenging but remain crucial in improving outcomes. In this report, we describe the diagnosis and management of a unique case of isolated, multifocal SMA vasculitis with concomitant superior mesenteric vein (SMV) thrombosis.

## Case presentation

A 45-year-old male was referred to the gastroenterology department for evaluation of an 18-month history of postprandial generalized abdominal pain associated with bloating. A three-month history of constipation was noted. He lost 20 pounds within the year. Famotidine and omeprazole were used without any relief. He did not use nonsteroidal anti-inflammatory drugs (NSAIDs) regularly. He did not abuse tobacco, alcohol, or recreational drugs. The patient reported no fever, night sweats, melena, rectal bleeding, appetite changes, headaches, chest pain, shortness of breath, oral ulcers, skin rashes, alopecia, and other symptoms suggestive of connective tissue disease. He had no pertinent medical history. Examination findings included cachexia.

**Figure 1:**
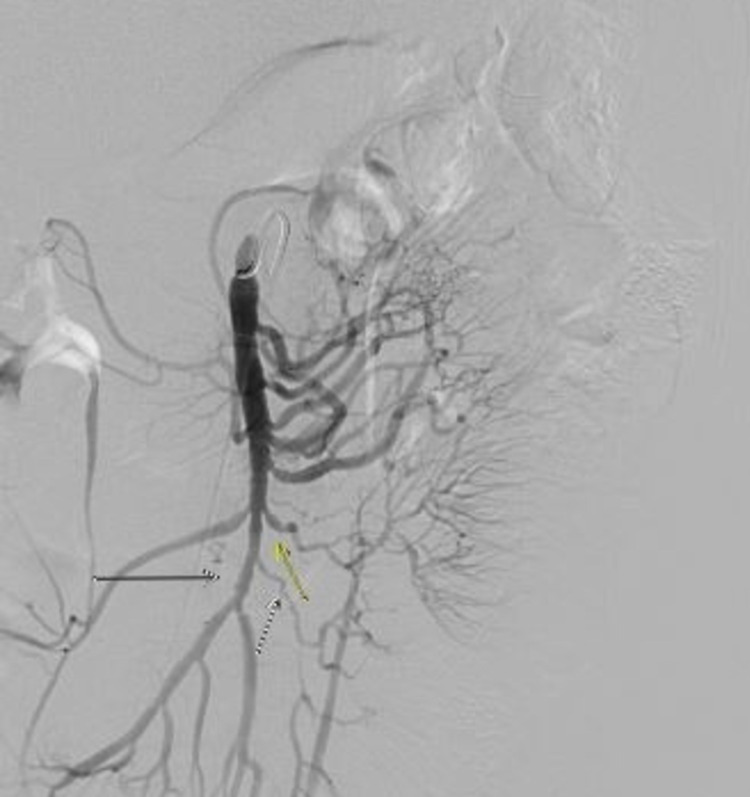
Abdominal angiography shows multifocal irregular beading and stenoses throughout the SMA distribution, indicating SMA vasculitis

**Figure 2:**
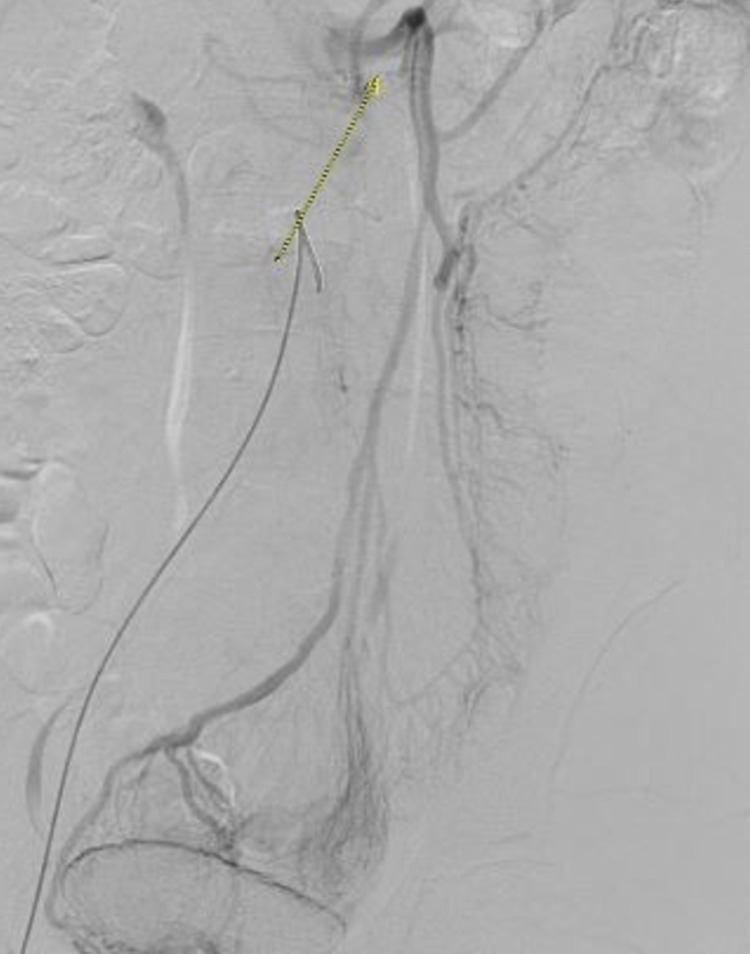
Abdominal angiography shows SMV occlusion with collaterals draining directly into the portal vein, suggesting SMV thrombosis

Initial laboratory data revealed decreased levels of vitamin B12, serum folate, ferritin, and albumin. Serum electrolytes, liver enzymes, creatinine, prothrombin time, and urinalysis were normal. An esophagogastroduodenoscopy and colonoscopy were normal. A computed tomography (CT) scan of the abdomen and pelvis with contrast revealed a suspected mesenteric arteriovenous malformation (AVM) associated with long segmental distal small bowel edema, likely ischemic from the associated steal phenomenon. Subsequent abdominal angiography revealed multifocal irregular beading and stenoses throughout the SMA distribution (Figure 1). The SMV was occluded with the venous drainage flowing from the inferior mesenteric artery distribution directly into the portal vein via mesenteric collaterals (Figure 2). The angiogram did not confirm any mesenteric AVM. Furthermore, arteries in the celiac, right, and left renal artery distributions appeared normal. Portal and splenic veins appeared patent. Other laboratory data included the following: erythrocyte sedimentation rate of 6 mm/hour, C-reactive protein of 1.1 mg/L, and negative viral hepatitis profile and human immunodeficiency virus tests. The antinuclear antibody titer, antineutrophil cytoplasmic antibodies, anti-double-stranded DNA, and complement levels were normal. Additional tests, including antiphospholipid antibody panel, T-SPOT TB test, rheumatoid factor (RF), myeloperoxidase antibody, anti-Pr-3 antibody, cyclic citrul peptide antibody IgG, cardiolipin antibody, and beta-2-glycoprotein IgA/IgG/IgM antibodies, were all noncontributory. Laboratory tests for hypercoagulable states were also unremarkable. Biopsy was not pursued after weighing the risks and benefits because angiogram was most consistent with vasculitis involving the SMA. In addition, all extensive laboratory investigations did not reveal any underlying connective tissue disorder as the etiology of vasculitis of the SMA. Our patient was ultimately diagnosed with isolated vasculitis limited to the SMA with concomitant SMV thrombosis.

We consulted a vascular surgeon and rheumatologist to assist with the management plan. Due to the multifocal involvement of the SMA, a decision was made for medical therapy alone. Oral prednisone at 1 mg/kg and aspirin at 81 mg daily were administered to manage the SMA vasculitis. Due to the high-dose steroid therapy, he was also given trimethoprim/sulfamethoxazole for primary prophylaxis against pneumocystis pneumonia (PCP) [2]. The SMV thrombosis in our patient appeared to be chronic with well-developed collateralization. Due to the extent of the thrombus and the findings of bowel edema with no identifiable risk factors for hypercoagulability, rivaroxaban was prescribed for only six months. He was also started on vitamin B12, folic acid, and iron supplements. Over the next few months, his postprandial abdominal pain and bloating resolved. He resumed a normal meal intake, returning to his baseline weight in six months. Follow-up CT angiography at six months showed resolution of the beading and stenoses of distal branches of the SMA. This imaging also noted improvement in perivascular stranding and soft tissue thickening associated with the SMA and its branches. In addition, the SMV appeared completely patent, indicative of SMV thrombosis resolution. Prednisone dosage was tapered at six months after initiation and was eventually stopped at nine months. In addition, anticoagulation with rivaroxaban was discontinued at six months. Our plan is to monitor the patient regularly for at least five years for either the recurrence of localized symptoms or the development of any systemic symptoms.

## Discussion

Although vasculitis of the GI tract often occurs in the presence of multisystem disease, the disease process and clinical course vary based on the size of the affected blood vessel. Large vessels are involved in systemic conditions such as giant cell arteritis and Takayasu arteritis. Medium-sized vessels are involved in Kawasaki disease and polyarteritis nodosa. Systemic lupus erythematosus, Churg-Strauss syndrome, Henoch-Schönlein purpura, and rheumatoid arthritis are associated with small vessel involvement [3]. Isolated mesenteric vasculitis without systemic involvement is a rare condition that may lead to bowel ischemia, intestinal edema, ileus, hemorrhage, or bowel stricture/perforation. Due to nonspecific symptoms, diagnosis based solely on clinical symptoms and biomarkers is challenging. Appropriate vascular imaging confirmed the diagnosis in our patient, precluding the need for biopsy.

Vasculitides of the gastrointestinal tract can be focal or diffuse. In the case of focal occlusive or aneurysmal vasculitis, either medical or surgical management is warranted [4,5]. However, surgical management is not optimal for multifocal disease, as was the case with our patient. Medical therapy with the use of steroids is the mainstay of treatment for such situations. Timely management led to successful clinical and radiographic outcomes in our patient. High clinical suspicion and early diagnosis are crucial for preventing the life-threatening complications associated with advanced mesenteric vasculitis. Progression to systemic vasculitis has been observed in nearly 25% of patients with localized mesenteric vasculitis over a five-year period [6]. Therefore, it is imperative that patients treated for localized mesenteric vasculitis be monitored for at least five years for the onset of systemic vasculitides.

Mesenteric vein thrombosis originating from a larger vein, such as the SMV, is usually due to compression at the site from an underlying disease, such as localized vasculitis in this case [7]. Slow-evolving SMV thrombus will often develop collateral veins, lowering the likelihood of intestinal infarction [8]. The present case is one in which isolated SMA vasculitis coexisted with SMV thrombosis. To our knowledge, there has been no prior report describing this combination.

## Conclusions

Isolated SMA vasculitis is a rare cause of mesenteric ischemia. Early diagnosis is challenging because of nonspecific clinical presentation. Herein, we have presented a case of an angiographically confirmed isolated vasculitis limited to the SMA with coexisting SMV thrombosis. Treatment with glucocorticoids alone resulted in the complete clinical and radiologic resolution of the regional vasculitis. This case highlights the importance of differentiating isolated vasculitis from systemic vasculitis as the former has a better prognosis in addition to avoiding the use of cytotoxic therapy and long-term glucocorticoids.
